# Response-shift effects in neuromyelitis optica spectrum disorder: estimating response-shift-adjusted scores using equating

**DOI:** 10.1007/s11136-020-02727-8

**Published:** 2021-01-05

**Authors:** Carolyn E. Schwartz, Roland B. Stark, Brian D. Stucky, Yuelin Li, Bruce D. Rapkin

**Affiliations:** 1 DeltaQuest Foundation, Inc., 31 Mitchell Road, Concord, MA 01742 USA; 2 Departments of Medicine and Orthopaedic Surgery, Tufts University Medical School, Boston, MA USA; 3 Departments of Psychiatry & Behavioral Sciences and Epidemiology and Biostatistics, Memorial Sloan Kettering Cancer Center, New York, NY USA; 4 Department of Epidemiology and Population Health, Albert Einstein College of Medicine, Bronx, NY USA

**Keywords:** Neuromyelitis optica spectrum disorder, Definitive neuromyelitis optica, Neurologic, Response shift, Clinical trial, Patient-reported outcome, Clinician-assessed outcome, Interpretation of change

## Abstract

**Background:**

In our companion paper, random intercept models (RIMs) investigated response-shift effects in a clinical trial comparing Eculizumab to Placebo for people with neuromyelitis optica spectrum disorder (NMOSD). RIMs predicted Global Health using the EQ-5D Visual Analogue Scale item (VAS) to encompass broad criteria that people might consider. The SF36™v2 mental and physical component scores (MCS and PCS) helped us detect response shift in VAS. Here, we sought to “back-translate” the VAS into the MCS/PCS scores that would have been observed if response shift had not been present.

**Methods:**

This secondary analysis utilized NMOSD clinical trial data evaluating the impact of Eculizumab in preventing relapses (*n* = 143). Analyses began by equating raw scores from the VAS, MCS, and PCS, and computing scores that removed response-shift effects. Correlation analysis and descriptive displays provided a more comprehensive examination of response-shift effects.

**Results:**

MCS and PCS crosswalks with VAS equated the scores that include and exclude response-shift effects. These two sets of scores had low shared variance for MCS for both groups, suggesting that corresponding mental health constructs were substantially different. The shared variance contrast for physical health was distinct only for the Placebo group. The larger MCS response-shift effects were found at end of study for Placebo only and were more prominent at extremes of the MCS score distribution.

**Conclusions:**

Our results reveal notable treatment group differences in MCS but not PCS response shifts, which can explain null results detected in previous work. The method introduced herein provides a way to provide further information about response-shift effects in clinical trial data.

## Introduction

Most clinicians recognize that patients adapt and show remarkable resilience to health-state changes [1], but work documenting such response-shift effects has largely focused on observational studies rather than clinical trials [2–4]. Researchers have long posited that response-shift effects would alter measured treatment differences in a clinical trial, due to differential effects of treatment versus placebo on quality-of-life (QOL) changes over time [5, 6]. In our companion paper [7], we investigated response-shift effects in a clinical trial comparing Eculizumab versus Placebo for people with neuromyelitis optica spectrum disorder (NMOSD). The pivotal trial documented remarkable effects of Eculizumab in preventing relapse [8], but subsequent analyses showed no such benefit on the SF-36™ mental component score (MCS) despite benefit on the SF-36™ physical component score (PCS) [9]. This lack of benefit on this evaluative outcome led us to hypothesize that response-shift effects were obfuscating treatment arm differences in mental health.

Consistent with theory, response shift was conceptualized as an epiphenomenon, and therefore it is inferred by the behavior of other measured variables [6, 10]. In our companion paper [7], we sought to adapt the Oort Structural Equation Modeling response-shift detection approach [11, 12] to the context of a small sample. Accordingly, we used random intercept modeling (RIM) [13] as we investigated and detected response-shift effects related to Treatment Arm and, more specifically, to the experience of relapse. The companion paper’s results suggested that the benefit of Eculizumab was underestimated in standard analyses [7]. These RIMs used VAS rather than MCS or PCS as an outcome. In order to explicate how response-shift effects may have clouded differences in MCS or PCS over the course of the trial, we sought to derive a method for communicating the VAS-based response-shift results in terms of MCS and PCS. This translation would move us closer to an estimate of response-shift adjusted change. We and others have long noted that response shift constitutes information, not ‘noise’ that should be removed [5, 6, 10, 14, 15]. In order, however, to clarify the response-shift effects in the trial data, one must contrast it with something [2]. This is why we are ‘back-translating’ the VAS scores into MCS/PCS scores with and without response-shift effects.

## Methods

### Sample and trial procedure

This secondary analysis utilized data from a randomized, double-blind, time-to-event trial evaluating the impact of Eculizumab in preventing relapses in 143 people with NMOSD. The interested reader is referred to the original pivotal trial [8] for details. The trial was conducted in accordance with the provision of the Declaration of Helsinki, the International Conference on Harmonization guidelines for Good Clinical Practice, and applicable regulatory requirements. The trial was approved by the institutional review board at each participating institution. All the patients provided written informed consent before participation.

### Measures

Analysis utilized information about Treatment Arm (i.e., Eculizumab vs. Placebo) as well as a three-level relapse variable defined in the companion publication [7]. Briefly, relapse was categorized into three groups: (1) no relapse; (2) clinician-reported relapse; and (3) adjudicated relapse. Whereas clinician-reported relapse was based on examination of patients with new symptoms and the determination that they met the protocol definition of on-trial relapse, adjudicated relapse also considered magnetic resonance (MRI) and optical coherence tomography (OCT) imaging data [8].

PRO data included the *EuroQOL 5-Dimension* 3-Level (EQ-5D-3L) Visual Analogue Scale (VAS) item [16]. This subjective, self-reported Global Health score ranged from 0 (worst imaginable health) to 100 (best imaginable health). The *Short-Form-36v2* (SF-36v2™) [17] is a generic evaluative measure of functional health that includes eight domain scores (general health, physical functioning, physical role performance, social functioning, emotional role performance, mental health, pain, vitality). Physical component score (PCS) and mental component score (MCS) are created from weighted sums of the eight domain scores [18]. The norm-based scoring system of the SF-36™ ranges from 0 to 100, with a normative mean of 50 and standard deviation of 10. Higher scores indicate better functional health.

### Statistical analysis

**Fig. 1:**
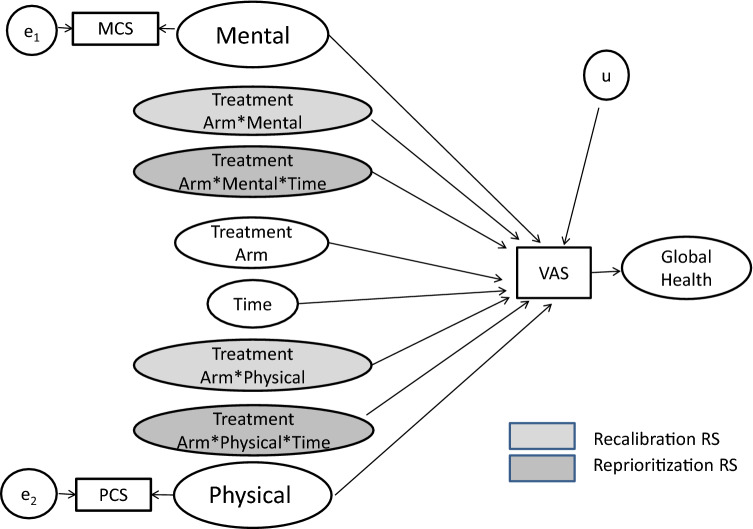
Conceptual model underlying response-shift analyses. Global Health was the latent variable of central focus in the analysis, and was operationalized as the EQ-5D VAS score. To capture how Treatment Arm group differentially emphasized physical and mental health overall and over time, *recalibration* was defined as a significant MCS (or PCS) * group interaction in predicting VAS (lightest grey shading); and *reprioritization* was defined as a significant MCS (or PCS) * group * time interaction predicting VAS (darker grey shading)

Figure 1 shows the conceptual model underlying our response-shift analyses reported in the companion paper [7]. The model shows that Global Health was the outcome variable of central focus in the analysis. This decision built on past research which demonstrated that there is a wider range of criteria that people might consider with a more global measure [14]. For example, someone’s score on a Global Health item could consider physical, mental, social, and spiritual aspects of their health, and we do not know which or in what balance. Measuring it by the EQ-5D VAS, we then sought to examine and model how physical and mental health were differently emphasized by catalyst group (e.g., Treatment Arm), and whether this differential emphasis changed over time. Briefly summarized, our approach used random intercept models (RIMs) [13] to test for response-shift effects by examining longitudinal differences in patterns of emphasis by catalyst group (Treatment Arm or, in a separate set of models, Relapse Group). *Recalibration* was defined as PCS and MCS differing by Treatment Arm (or Relapse Group) in their ability to explain EQ-5D VAS scores (e.g., significant MCS*Treatment Arm interaction in predicting VAS). *Reprioritization* was defined as such dynamics changing over time (e.g., interaction of Treatment Arm* MCS*Time in predicting VAS). *Reconceptualization* was addressed in a separate series of RIMs predicting each QOL domain from catalyst group after adjusting for the other eight domains [7].

The present work sought to estimate MCS and PCS scores at baseline and at end of study by Treatment Arm, with and without response-shift effects. To “translate” the MCS and PCS scores from the predicted value of VAS with and without response-shift effects, we utilized the classical-test theory method of equipercentile ranking to equate scores [19–21]. In this approach, a crosswalk is created that links scores on two or more PROs by equating scores that represent the same percentile ranking in the sample [20–22]. In our case, the VAS score was linked with the MCS and PCS scores. We began by creating a linking function between the raw VAS and MCS or PCS scores at all time points (i.e., the specific MCS or PCS scale score reflecting the same percentile ranking for a given raw VAS value). These ‘raw’ scores would include response-shift effects.

**Fig. 2:**
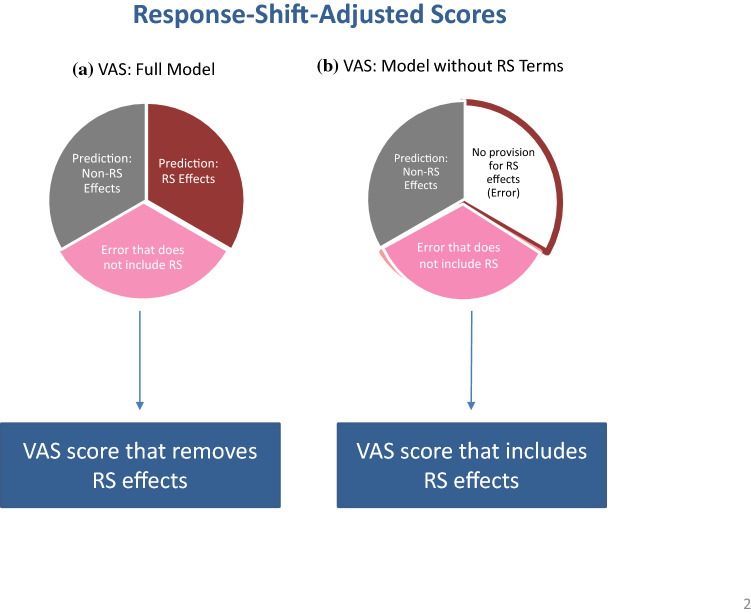
Pie chart illustrating how VAS scores without (**a**) and with (**b**) response-shift (RS) effects are estimated. We sought to compare VAS scores with and without response-shift effects in the model. Accordingly, we wanted to estimate a VAS score that removed variance related to recalibration and reprioritization response shifts (i.e., group-by-MCS [or PCS] and group-by-MCS [or PCS]-by-time). This figure shows the logic using two pie charts, not drawn to scale, of the variance components in the random intercept models. In order to obtain the desired score, we estimated VAS in two ways: with response-shift terms in the model (the full model as shown in **a**) and without (as shown in **b**). In the model without response shift terms, what would be in the response shift terms is left in the error variance (**b**). In the model with response shift terms, response shift is removed from the error variance (**a**). In order to obtain VAS scores with and without response-shift effects—the score that would be as if there were no response shift—we took the predicted score without response shift and added in the error term from the full model (i.e., the error term without response shift)

Our goal for conducting this crosswalk was to compare VAS scores with and without response-shift effects using four random intercept models (Table 1). Model 1 included fixed effects representing recalibration and reprioritization response-shift effects (i.e., group-by-MCS (or PCS) and group-by-MCS (or PCS)-by-time). Model 1.a then yielded the estimated VAS scores with the response-shift effects. Next, in model 2, the response-shift terms were excluded to yield estimated VAS scores without response shift. Model 2 effectively presumed that VAS ratings were attributable solely to MCS/PCS and treatment, but not to recalibration and reprioritization in the interaction terms. Hence, model 2 yielded VAS scores that removed variance related to recalibration and reprioritization response shifts. The final model (model 3) was constructed by taking the estimated VAS without response-shift effects (predicted VAS value from model 2) and then adding the residual from model 1 to account for idiosyncratic variabilities under response shift. The diagrams in Fig. 2 represent the variances accounted for in these models (Fig. 2a for model 1; 2b for model 3).

**Table 1:** Method for adjusting scores specifically for response shift

	Model name	Terms in the model	Comment
1	Full model	VAS_it_ = *β*_0_ + *β*_1_ PCS_i_ + *β*_2_ Tx_i_ + *β*_3_ PCS_i_*Tx_i_ + *β*_4_ PCS_i_*Tx_i_*Time_t_ + *b*_i_ + *ε*1_it_, where *b*_i_ ~ *N*(0, *σ*_1_^2^) as random intercepts per person, normally distributed with mean 0 and variance *σ*_1_^2^; and *ε*1_it_ represents idiosyncratic residuals when response-shift terms are included	Includes response-shift fixed-effect terms *β*_1_, *β*_2_, *β*_3_, and *β*_4_ (i.e., significant interactions) and an overall intercept *β*_0_. The residual of model 1, *ε*1_it_, excludes response-shift variance as well as main effect
1a	Predicted score from full model	*E*(VAS_it_) = *β*_0_ + *β*_1_ PCS_i_ + *β*_2_ Tx_i_ + *β*_3_ PCS_i_*Tx_i_ + *β*_4_ PCS_i_*Tx_i_*Time_t_ + *b*_i_	What one obtains when the statistical program saves predicted score
2	Predicted score from a reduced model (response-shift fixed effects excluded)	*E*(VAS_it_) = *β*_0_ + *β*_1_ PCS_i_ + *β*_2_ Tx_i_ + *b*_i_ + *ε*2_it_, where *b*_i_ ~ *N*(0, *σ*_2_^2^), as random intercepts per person	Predicted score of model 2: excludes RS variance and residual variance
3	Estimated score removing response-shift effects: Predicted score from a reduced model (response-shift fixed effects excluded)	VAS_it_ = *β*_0_ + *β*_1_ PCS_i_ + *β*_2_ Tx_i_ + *b*_i_ + *ε*1_it_, where *b*_i_ ~ *N*(0, *σ*_1_^2^), as random intercepts per person in model 2	Add the residual from the full model 1 with the predicted score from the main-effects-only model 2 That is the original VAS score adjusted only for response-shift interaction terms

In addition to the abovementioned crosswalks, comparisons between scores were investigated using Pearson correlation coefficients. Cohen’s criteria for magnitude of effect sizes were used [23]. To clarify what ranges of MCS (or PCS) revealed larger response-shift effects over study follow-up, bar charts were used to display differences between scores that included and excluded response-shift effects at baseline and end of study by Treatment Arm.

Statistical analyses were implemented using IBM SPSS version 26 [24] and Microsoft Office 365 Excel® [25].

## Results

### Sample

The study sample included 143 people, of whom 107 had definitive neuromyelitis optica and 36 had NMO Spectrum Disorder. Two thirds of the sample was on Eculizumab and one third on placebo, and the sample displayed high levels of treatment adherence. The predominantly female sample had a mean age of 44 and a mean age of diagnosis of 41. See [7] for a full description of study sample demographics. Each patient had between three and 23 clinician visits during the trial, and each spent between two and 30 months under study.

### Crosswalks

**Fig. 3:**
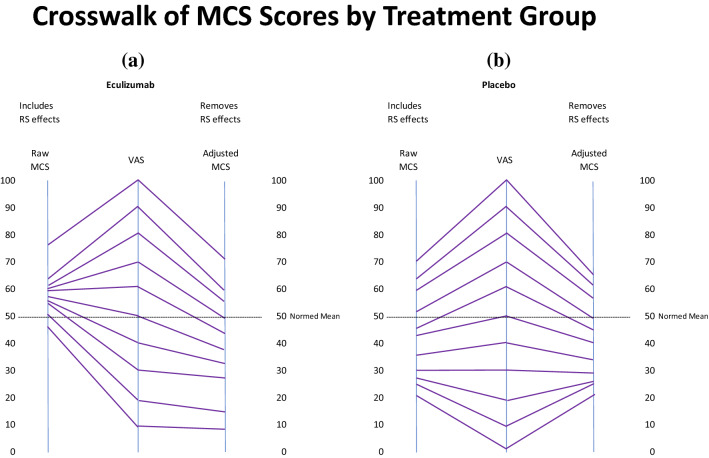
Crosswalk between VAS and MCS scores, including and excluding response-shift effects by Treatment Group. This graphic presentation of the crosswalk between the VAS and the MCS scores illustrates how response-shift effects may alter the apparent treatment group differences in the clinical trial data. The left-most crosswalk shows the linkage for the Eculizumab patients (**a**), showing that the MCS scores that include response-shift effects (on the left) have a more truncated range and are higher than the population norm compared to those that exclude response-shift effects (on the right). The right-most crosswalk shows the linkage for Placebo patients (**b**), exhibiting a similar range and similar linked scores for MCS scores that include and exclude response-shift effects

A graphic presentation of the *crosswalk* between the VAS and the MCS scores that include and exclude response-shift effects is shown in Fig. 3a, b. The left-most crosswalk shows the linkage for the Eculizumab patients (3a), and the right-most shows it for Placebo patients (3b). For Eculizumab patients, the MCS scores that include response-shift effects have a more truncated range. The lowest scores are very close to the population norms and have a range of only 30. In contrast, the MCS scores that exclude response-shift effects have more than twice that range. The Placebo group’s crosswalk does not show such differences, exhibiting a similar range and similar linked scores for MCS scores that include and exclude response-shift effects.

**Fig. 4:**
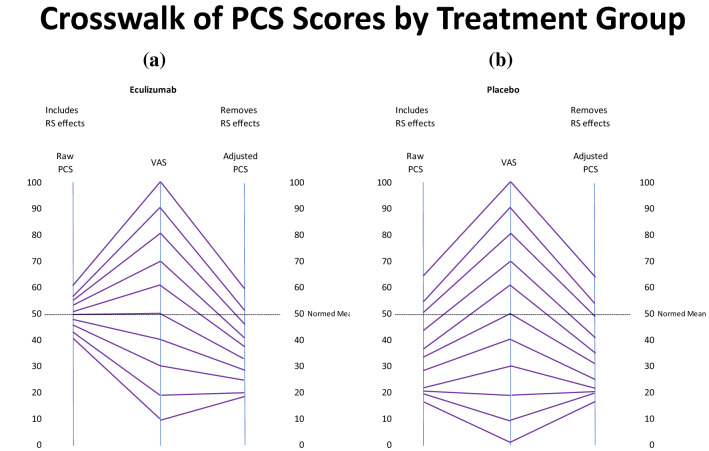
Crosswalk between VAS and PCS scores, including and excluding response-shift effects by Treatment Group. This graphic presentation of the crosswalk between the VAS and the PCS scores illustrate a similar pattern to the MCS crosswalk. Eculizumab patients (**a**) show a more truncated distribution for the Eculizumab group’s scores that include response-shift effects. In contrast to this group’s MCS scores, the PCS scores that include response-shift effects reflect worse physical functioning compared to population norms. The Placebo group’s crosswalk (**b**) exhibits a similar range and similar linked scores for PCS scores that include and exclude response-shift effects

Figure 4a, b display the PCS crosswalks, again with the left-most crosswalk showing the linkage for the Eculizumab patients (4a), and the right-most showing it for Placebo patients (4b). The pattern is similar to the above MCS pattern, with a more truncated distribution for the Eculizumab group’s scores that include response-shift effects. In contrast to this group’s MCS scores, the PCS scores that remove response-shift effects reflect worse physical functioning compared to population norms. Compared to the Placebo group’s MCS pattern, the Placebo group’s crosswalk exhibits a similar range and similar linked scores for PCS scores that include and exclude response-shift effects.

These crosswalks utilize all available data points, in particular multiple points per patient. They thus provide a more robust indicator of scores at a given percentile. Based on this illustration, one might surmise that response-shift effects alter our best estimates of treatment group differences. The crosswalks are, however, idealized illustrations of the correspondence between scores that include and exclude response-shift effects. The crosswalks are good for creating a link between all scores, but are not helpful for characterizing the magnitude of the response-shift effect altogether. Further subgrouping by-time point would be necessary to characterize these response-shift effects and their impact.

### Associations between scores that include and exclude response-shift effects

Table 2 shows the correlations between VAS, MCS, and PCS scores that include and exclude response-shift effects for the overall sample, and by Treatment Arm. Here, *smaller* correlations signal greater divergence and thus larger response-shift effects. These findings shows large effect-size correlations between the two types of VAS scores, overall and across groups, and large effect-size correlations for the PCS overall and for the Eculizumab group. In contrast, the correlations for MCS were medium-effect-size overall and for both groups, and for PCS for the Placebo group. In other words, overall VAS and PCS scores with response-shift effects explain 83% and 35% of the variance in scores without them, respectively. In contrast, for MCS scores overall, this number is closer to 14%. This contrast corroborates our results showing greater response-shift effects for MCS.

**Table 2:** Correlations between scores including and excluding response-shift effects

Score	Overall (*n* = 1409)	Eculizumab (*n* = 1040)	Placebo (*n* = 368)
VAS	**0.91**	**0.92**	**0.89**
MCS	*0.38*	*0.37*	*0.37*
PCS	**0.59**	**0.62**	*0.46*

Bold and italics indicated whether the correlation coefficient is a medium or large effect size, respectively, using Cohen’s criteria

*n* = number of visits (> 1 per patient)

### Baseline versus end-of-study differences in magnitude of response-shift effects

**Fig. 5:**
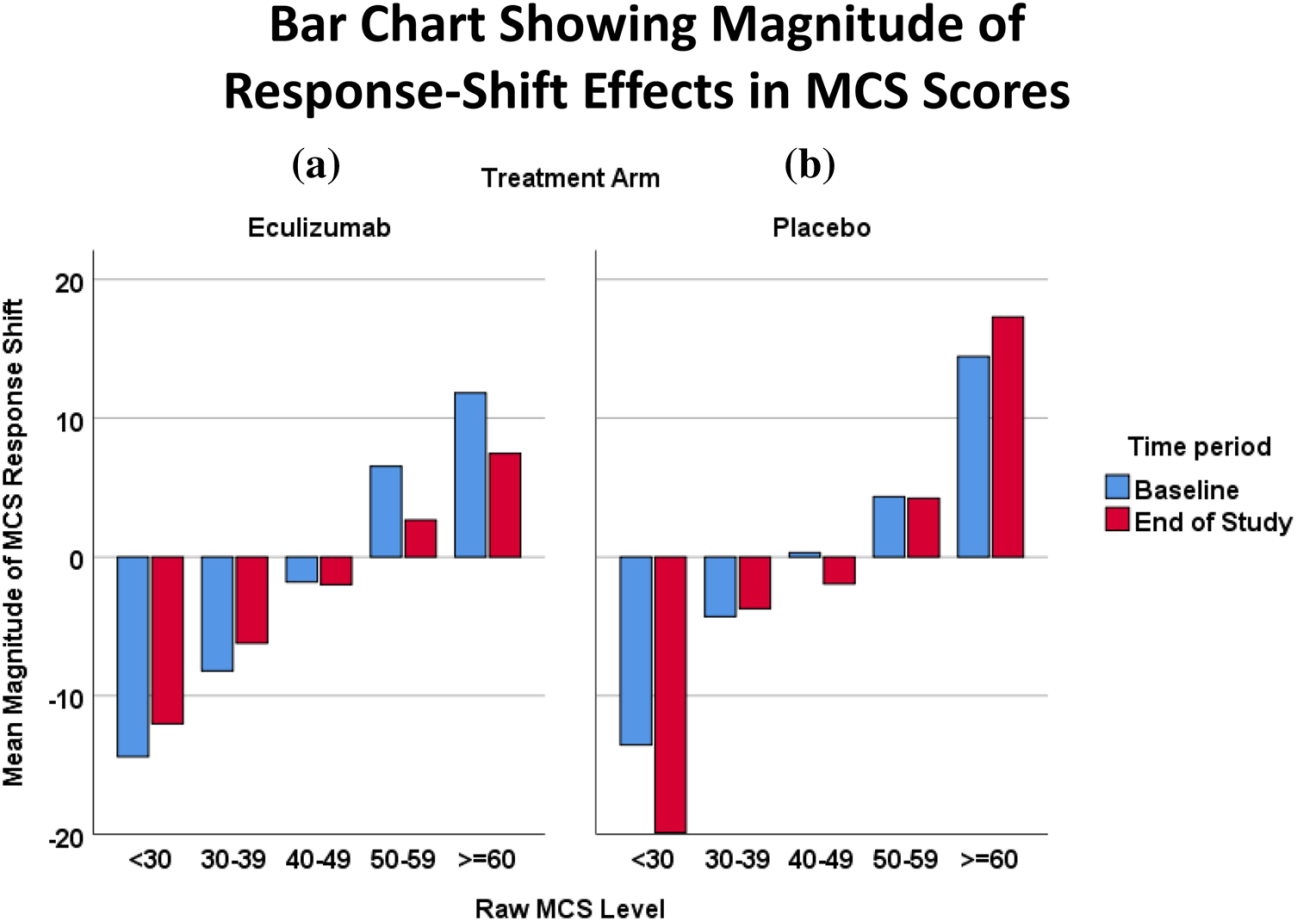
Bar chart showing magnitude of response-shift effects in MCS scores. Bar charts illustrate MCS scores excluding response-shift effects at baseline versus end of study for Eculizumab (**a**) and Placebo (**b**) patients. This plot illustrates that the larger MCS response-shift effects were found at end of study for Placebo as compared to Eculizumab, and the effects are more prominent at extreme ends of the spectrum (i.e., very low and very high MCS scores). For the Eculizumab patients, response-shift effects are smaller at end of study than at baseline almost at every level of MCS raw scores

Figures 5a and b display bar charts showing MCS scores excluding response-shift effects at baseline versus end of study for Eculizumab (5a) and Placebo (5b) patients. This plot illustrates that the larger MCS response-shift effects were found at end of study for Placebo as compared to Eculizumab, and the effects are more prominent at extreme ends of the spectrum (i.e., for patients with very low and very high raw MCS scores). In contrast, for the middle three categories of MCS raw scores, the response-shift differences by-time period are negligible for the Placebo group. For the Eculizumab patients, response-shift effects are smaller at end of study than at baseline at almost every level of MCS raw scores.

**Fig. 6:**
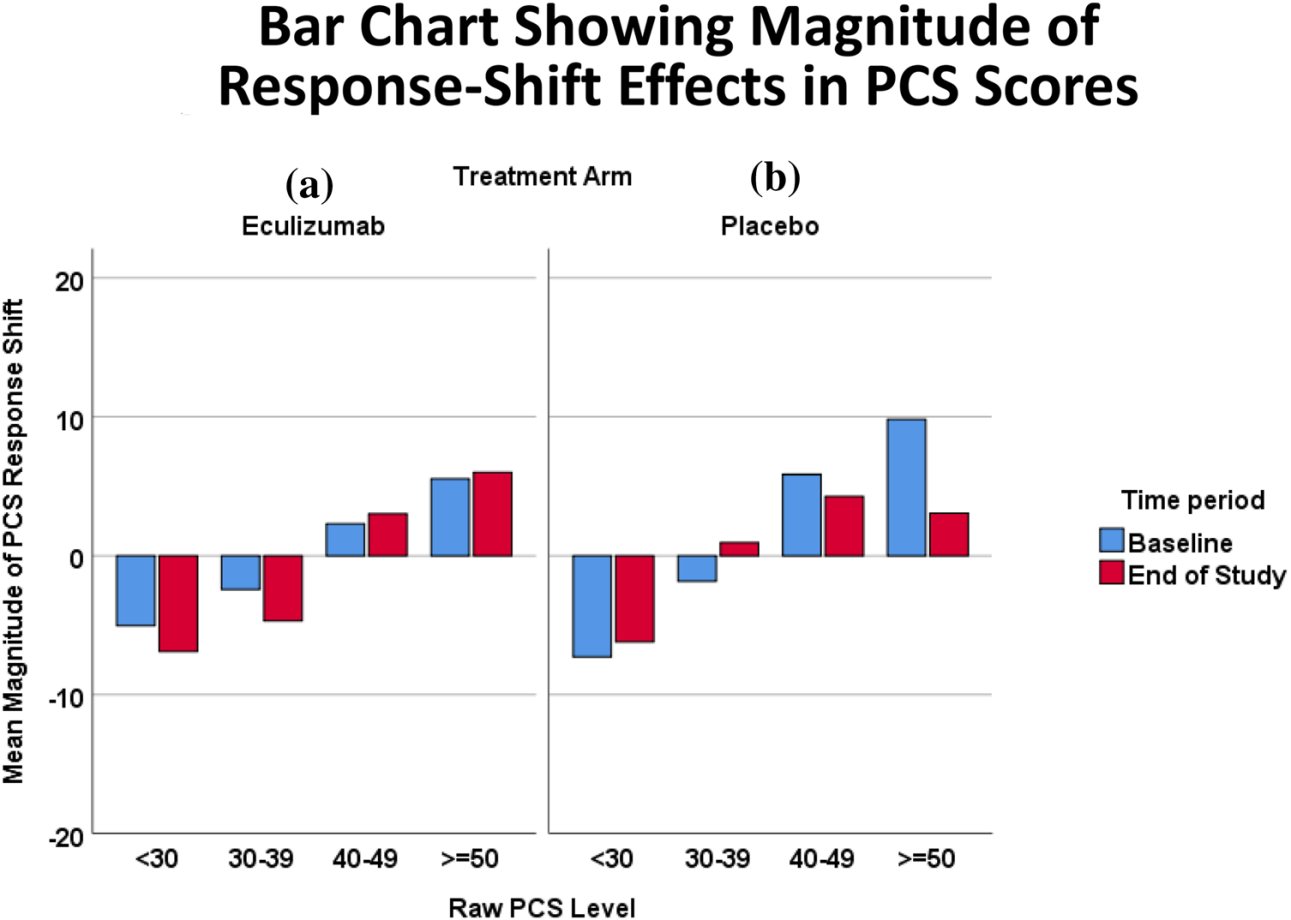
Bar chart showing magnitude of response-shift effects in PCS scores. Bar charts illustrate PCS scores excluding response-shift effects at baseline versus end of study for Eculizumab (**a**) and Placebo (**b**) patients. Overall, the response-shift effects for PCS were smaller across groups than for MCS, and the larger effects were for high PCS scores for the Placebo patients at baseline. For the Eculizumab patients, the largest response-shift effects were at extreme low PCS scores at end of study

Figure 6a and b display bar charts showing PCS scores excluding response-shift effects at baseline versus end of study for Eculizumab (6a) and Placebo (6b) patients. This plot illustrates that for both groups, the response-shift effects for PCS were considerably smaller than for MCS. Relatively large effects occurred for high raw PCS scores for the Placebo patients at baseline.

## Discussion

We present a novel method for deriving response-shift-adjusted scores from the RIM response-shift detection method described in our companion paper [7]. By using equating to generate crosswalks between scores that include and exclude response-shift effects, we are able to clarify how such effects could have altered the apparent Treatment Group differences on MCS in the clinical trial analyses [8]. As noted in our companion paper [26], published trial results likely underestimated Eculizumab vs. Placebo differences in mental health due to recalibration and reconceptualization. Thus, the difference in mental health between the Eculizumab and Placebo patients was likely wider than it appeared.

Our analyses document that Eculizumab patients’ MCS and PCS scores that include response-shift effects have a more truncated range, which generally makes them look better off than scores that remove response-shift effects. In contrast, Placebo patients’ crosswalks for both MCS and PCS exhibit similar ranges and similar linked scores whether including or excluding response-shift effects. Further investigation revealed, however, that the Placebo patients had the larger MCS response-shift effects at end of study, at very low and very high ends of the raw score distribution, whereas the Eculizumab patients’ response-shift effects were larger at baseline than at end of study. This would suggest that the Placebo patients, who experienced the vast majority of the relapses, engaged in mentalhealth response shifts after the relapse (i.e., at end of study), thereby enabling them to maintain homeostasis in mental health. These discrepancies could thus work to make analyses of group MCS differences at end of study appear to yield null results. In contrast, the PCS scores generally do not exhibit large differences between scores including and excluding response-shift effects.

As noted in our companion paper, our findings likely reflect the ‘shadow’ of response shift, inferred by the behavior of examined interactions and unique variance explained rather than characterized more directly. Our analyses suggest that response-shift effects were most prominent for MCS for both groups. Based on Fig. 6b as well as Table 2, there was some indication that such effects occurred to a lesser degree for PCS, and mainly for the Placebo group. In other words, people on placebo, who in this study had a much higher rate of relapse, were thinking differently about health due to their experiences: they emphasized the physical more and the mental less than did the Eculizumab group. Based on both groups’ strikingly low shared variance between MCS scores including and excluding response-shift effects (*R*^2^ = 0.14 for both groups), the construct of mental health reflected in the two sets of scores must be substantially different. In contrast, the construct of physical health tapped by the two sets of PCS scores is relatively similar for Eculizumab patients but different for Placebo patients (*R*^2^ = 0.38 vs. 0.21, respectively). It should also be noted that the number of visits and follow-up time are related strongly with relapse status. Indeed, the relapse analyses presented in the companion paper [26] suggest that the response-shift effects found in the treatment group comparisons are even stronger when patients are grouped by relapse status.

While the present work has the advantage of providing more insight into the impact of response shift on evaluative mental- and physical- health indicators, its limitations must be acknowledged. First and foremost, our crosswalk approach utilized VAS scores from the RIMs that captured recalibration and reprioritization but not reconceptualization response-shift effects. These latter were captured in a separate series of RIMs examining unique group variance explained by SF-36™ domain scores and VAS, and were not feasible to include in the crosswalk method. Response-shift effects may thus be underestimated by leaving out reconceptualization in the translated scores. Further, the available data were drawn from the formal clinical trial, not the extension- study data. Since in the formal trial patients only contributed one additional data point after relapse (i.e., at end of study), the response-shift effects may be attenuated as compared to longer-term follow-up after relapse. Another limitation involves adding the residuals from the full model into model 2 to yield the estimated scores after removing response-shift effects. Residuals are random errors produced by a model. Adding the residuals as is, we treated them as fixed quantities, which is incongruent with the notion of random errors. This was a crude but pragmatic way to account for idiosyncratic variabilities after the response-shift effects were accounted for. Future research may devise a statistically more principled method to estimate these individual variabilities. For example, a term representing random error could be estimated independently using Monte Carlo simulation, bootstrapped, and added into model 3 to determine the range of results (i.e., confidence interval for effects as reported in Figs. 5 and 6). More straightforwardly, our analyses rest on certain assumptions. It assumes that specific values of residuals and person-specific random intercepts remain invariant when the residuals from one model are added into another. However, this may be viewed as a crude but pragmatic way to parse out response-shift effects from the measurements. There is also the possibility of misspecification of our RIM, which could affect our findings. Finally, a contrarian may raise the point that the evidence of response shift in MCS for those in the extreme groups (and not for the middle three categories) could represent a regression toward the mean. In fact, the pattern was not symmetrical, which would lend more support to a response-shift rather than statistical-artifact interpretation.

In summary, the method introduced herein provides a way to glean further information about response-shift effects in clinical trial data. Our results reveal Treatment Group differences in MCS response shifts, which have important implications for null results detected in previous work [8]. It is our hope that the new applications of methods presented in both this paper and its companion will open new pathways for clinical research on new drug treatments and patient resilience.

## Data Availability

The study data are confidential and thus not able to be shared.

## Abbreviations

MCS: Mental component score of the SF-36™
NMOSD: Neuromyelitis optica spectrum disorder
PCS: Physical component score of the SF-36™
PRO: Patient-reported outcome
SF-36v2™: Short-Form-36v2
QOL: Quality of life
VAS: Visual Analogue Scale indicator of Global Health on the EQ-5D

## References

[1] Wilson IB (1999). Clinical understanding and clinical implications of response shift. Social Science and Medicine.

[2] Schwartz CE, Ahmed S, Sawatsky R, Sajobi T, Mayo N, Finkelstein JA, Lix L, Verdam MGE, Oort FJ, Sprangers MAG (2013). Guidelines for secondary analysis in search of response shift. Quality of Life Research.

[3] Schwartz CE, Bode R, Repucci N, Becker J, Sprangers MAG, Fayers PM (2006). The clinical significance of adaptation to changing health: A meta-analysis of response shift. Quality of Life Research.

[4] Sajobi TT, Brahmbatt R, Lix LM, Zumbo BD, Sawatzky R (2018). Scoping review of response shift methods: Current reporting practices and recommendations. Quality of Life Research.

[5] Sprangers MAG, Schwartz CE (1999). Integrating response shift into health-related quality of life research: A theoretical model. Social Science and Medicine.

[6] Rapkin BD, Schwartz CE (2004). Toward a theoretical model of quality-of-life appraisal: Implications of findings from studies of response shift. Health and Quality of Life Outcomes.

[7] Schwartz, C. E., Stark, R. B., Stucky, B. D., Li, Y., & Rapkin, B. D. (2020) Response-shift effects in Neuromyelitis Optica Spectrum Disorder: Estimating response-shift-adjusted scores using equating. Under review 10.1007/s11136-020-02727-8 PMC8068715 33398520

[8] Pittock SJ, Berthele A, Fujihara K, Kim HJ, Levy M, Palace J (2019). Eculizumab in aquaporin-4–positive neuromyelitis optica spectrum disorder. New England Journal of Medicine.

[9] Berthele, A., Pittock, S. J., Fujihara, K., Kim, H. J., Levy, M., Palace, J., et al. (2019). Impact of eculizumab on reported quality of life in patients with aquaporin-4 antibody-positive neuromyelitis optica spectrum disorder: Findings from the PREVENT study. In: European Committee for Treatment and Research in Multiple Sclerosis (ECTRIMS), Stockholm, Sweden, September 11–13, 2019.

[10] Rapkin BD, Schwartz CE (2019). Advancing quality-of-life research by deepening our understanding of response shift: A unifying theory of appraisal. Quality of Life Research.

[11] Oort FJ (2005). Using structural equation modeling to detect response shifts and true change. Quality of Life Research.

[12] Oort FJ, Visser MRM, Sprangers MAG (2005). An application of structural equation modeling to detect response shifts and true change in quality of life data from cancer patients undergoing invasive surgery. Quality of Life Research.

[13] Laird NM, Ware JH (1982). Random-effects models for longitudinal data. Biometrics.

[14] Schwartz CE, Rapkin BD (2004). Reconsidering the psychometrics of quality of life assessment in light of response shift and appraisal. Health and Quality of Life Outcomes.

[15] Barclay-Goddard R, Epstein JD (2009). Response shift: A brief overview and proposed research priorities. Quality of Life Research.

[16] Foundation ER (2018). EQ-5D-3L User Guides.

[17] Ware JE, Bayliss MS, Rogers WH, Kosinski M, Tarlov AR (1996). Differences in 4-year health outcomes for elderly and poor, chronically ill patients treated in HMO and fee-for-service systems. Results from the Medical Outcomes Study. JAMA.

[18] Ware JE, Kosinski M, Dewey JE (2000). How to score version 2 of the SF-36 health survey (standard & acute forms).

[19] Kolen MJ, Brennan RL (1995). Test equating: Methods and practices.

[20] Wu AW, Huang I-C, Gifford AL, Spritzer KL, Bozzette SA, Hays RD (2005). Creating a crosswalk to estimate AIDS Clinical Trials Group quality of life scores in a nationally representative sample of persons in care for HIV in the United States. HIV Clinical Trials.

[21] Fong TG, Fearing MA, Jones RN, Shi P, Marcantonio ER, Rudolph JL, Yang FM, Kiely DK, Inouye SK (2009). Telephone interview for cognitive status: Creating a crosswalk with the Mini-Mental State Examination. Alzheimer’s & Dementia.

[22] Schwartz CE, Bode RK, Quaranto BR, Vollmer T (2012). The symptom inventory disability-specific short forms for multiple sclerosis: Construct validity, responsiveness, and interpretation. Archives of Physical Medicine and Rehabilitation.

[23] Cohen J (1992). A power primer. Psychological Bulletin.

[24] IBM (2019). IBM SPSS statistics for windows.

[25] Microsoft® (2020). Excel®.

[26] Schwartz, C. E., Stark, R. B., & Stucky, B. D. (2020). Response-shift effects in neuromyelitis optica spectrum disorder: A secondary analysis of clinical-trial data.* Quality of Life Research*. 10.1007/s11136-020-02707-y. 10.1007/s11136-020-02707-y PMC8068626 33269417

